# Aging Equity and Institutional Frameworks: The Role of Policy and Applied Interventions

**DOI:** 10.1093/geroni/igaf122.1093

**Published:** 2025-12-31

**Authors:** Sarah Szanton

**Affiliations:** Johns Hopkins University, Baltimore, Maryland, United States

## Abstract

Aging equity is shaped by lifecourse institutional frameworks, including government policies, social structures, and healthcare systems, that influence social determinants of health, access to care, and the overall well-being of older adults. Research demonstrates that systemic inequities—particularly those rooted in historical injustices such as redlining—compound over the life course, leading to inequities in not just life span but health span. This session will examine how policy-driven approaches can address these disparities and promote sustainable, equitable aging solutions. A critical focus of this discussion is the CAPABLE (Community Aging in Place—Advancing Better Living for Elders) program, an evidence-based intervention designed to enhance functional independence, reduce healthcare costs, and improve quality of life among older adults. CAPABLE integrates a nurse, an occupational therapist, and a home repair specialist to support self-directed goals for aging in place. Longitudinal research has demonstrated a 7-10x return on investment, primarily through reductions in hospitalization and nursing home admissions. We will also touch on a new model called Neighborhood Nursing, which provides a nurse and community health worker, block-by-block, to all people. By integrating applied research into aging policy frameworks, findings illustrate how structural innovations can drive inclusion, reduce disparities, and sustain progress in aging equity.

